# (1′*S*,4′*S*)-5-(2,5-Dimethyl­phen­yl)-4′-meth­oxy-6-oxa-3-aza­spiro­[bicyclo­[3.1.0]hexane-2,1′-cyclo­hexa­n]-4-one

**DOI:** 10.1107/S1600536813005138

**Published:** 2013-02-28

**Authors:** Xing-Rui He, Bing-Rong Xu, Jing-Li Cheng, Jin-Hao Zhao

**Affiliations:** a College of Pharmaceutical Science, Zhejiang University of Technology, Hangzhou 310032, People’s Republic of China; b Institute of Pesticide and Environmental Toxicology, Zhejiang University, Hangzhou 310029, People’s Republic of China

## Abstract

In the title compound, C_18_H_23_NO_3_, the cyclo­hexane ring has a chair conformation. The oxirane plane (OCC) makes a dihedral angle of 76.15 (13)° with that of the pyrrolidine ring to which it is fused. The mean plane of the cyclo­hexane ring and the benzene ring are almost normal to the pyrrolidine ring, with dihedral angles of 88.47 (8) and 77.85 (8)°, respectively. In the crystal, mol­ecules are linked *via* pairs of N—H⋯O hydrogen bonds, forming inversion dimers. These dimers are linked *via* pairs of C—H⋯O hydrogen bonds, forming chains along the *a*-axis direction.

## Related literature

For the pesticide spiro­tetra­mat, the central unit of the title compound, see: Fischer & Weiss (2008 ▶); Maus (2008 ▶). For structures of spiro­tetra­mat derivatives, see: Fischer *et al.* (2010 ▶). For the metabolic transformation of spiro­tetra­mat, see: Bruck *et al.* (2009 ▶)
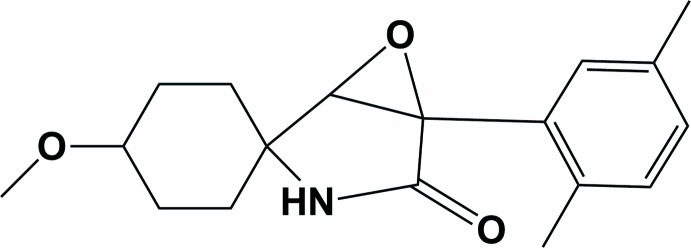


## Experimental

### 

#### Crystal data

C_18_H_23_NO_3_
*M* *_r_* = 301.37
Monoclinic, 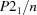

*a* = 9.1932 (4) Å
*b* = 9.8139 (4) Å
*c* = 17.6979 (7) Å
β = 91.198 (1)°
*V* = 1596.38 (11) Å^3^
*Z* = 4
Mo *K*α radiation
μ = 0.09 mm^−1^
*T* = 296 K
0.53 × 0.38 × 0.36 mm

#### Data collection

Rigaku R-AXIS RAPID/ZJUG diffractometer
Absorption correction: multi-scan (*ABSCOR*; Higashi, 1995 ▶) *T*_min_ = 0.946, *T*_max_ = 0.970
15333 measured reflections
3629 independent reflections
2407 reflections with *I* > 2σ(*I*)
*R*_int_ = 0.040

#### Refinement

*R*[*F* ^2^ > 2σ(*F* ^2^)] = 0.045
*wR*(*F* ^2^) = 0.122
*S* = 1.00
3629 reflections
203 parameters
H-atom parameters constrained
Δρ_max_ = 0.26 e Å^−3^
Δρ_min_ = −0.23 e Å^−3^

### 

Data collection: *PROCESS-AUTO* (Rigaku, 2006 ▶); cell refinement: *PROCESS-AUTO*; data reduction: *CrystalStructure* (Rigaku,2007 ▶); program(s) used to solve structure: *SHELXS97* (Sheldrick, 2008 ▶); program(s) used to refine structure: *SHELXL97* (Sheldrick, 2008 ▶); molecular graphics: *ORTEP-3 for Windows* (Farrugia, 2012 ▶); software used to prepare material for publication: *WinGX* (Farrugia, 2012 ▶).

## Supplementary Material

Crystal structure: contains datablock(s) global, I. DOI: 10.1107/S1600536813005138/su2559sup1.cif

Supplementary material file. DOI: 10.1107/S1600536813005138/su2559Isup2.cdx

Structure factors: contains datablock(s) I. DOI: 10.1107/S1600536813005138/su2559Isup4.hkl

Supplementary material file. DOI: 10.1107/S1600536813005138/su2559Isup4.cml

Additional supplementary materials:  crystallographic information; 3D view; checkCIF report

## Figures and Tables

**Table 1:** Hydrogen-bond geometry (Å, °)

*D*—H⋯*A*	*D*—H	H⋯*A*	*D*⋯*A*	*D*—H⋯*A*
N1—H1⋯O3^i^	0.86	2.25	3.0760 (17)	160
C9—H9*A*⋯O2^ii^	0.97	2.56	3.413 (2)	147

Symmetry codes: (i) 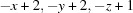; (ii) 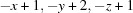.

**Fig. 1.**
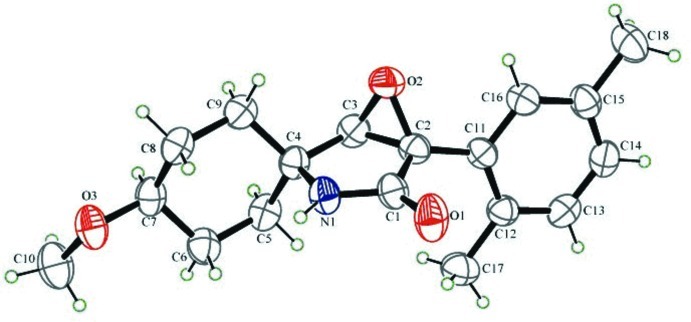
The molecular structure of the title molecule, with atom labelling. Displacement ellipsoids are drawn at the 40% probability level.

**Fig. 2.**
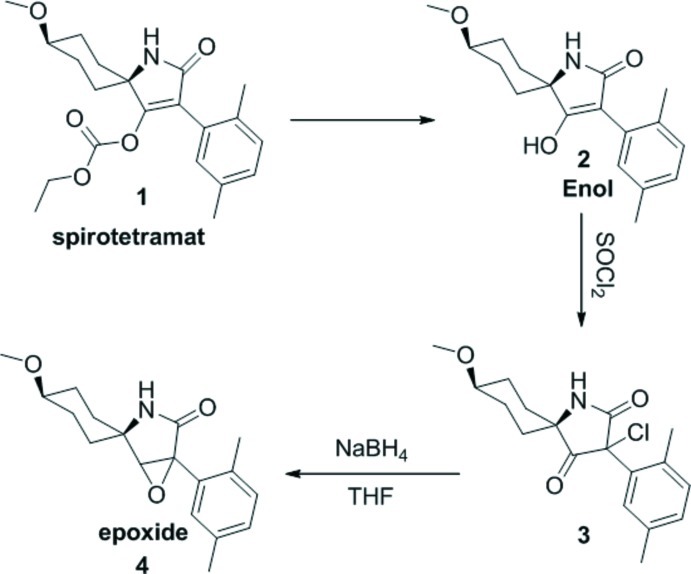
Reaction scheme.

## supplementary crystallographic information

### Comment

Spirotetramat is a new systemic insecticide which belongs chemically to the class of spirocyclic tetramic acid derivatives and be developed by Bayer CropScience AG (Fischer *et al.*, 2008; Maus, 2008). Recently, it has been found that when spirotetramat was introduced into plants or animals, it was hydrolysed to its enol form and as a weak acid this metabolite can move acropetally and basipetally with small log *P* (Bruck *et al.*, 2009). That is, the excellent bioactivities of spirotetramat maybe caused by this metabolite, which stimulated our interest in the synthesis of some novel analogues of this metabolite. We have designed a new simple route to synthesized the title compound and report herein on its crystal structure.

The molecular structure of the title molecule is shown in Fig. 1. The cyclohexane ring adopts a chair conformation; atoms C5, C6, C8 and C9 lie in a plane with atoms C4 and C7 deviating by 0.676 (4) and -0.664 (0) Å, respectively. The oxirane plane (O2/C2/C3) makes a dihedral angle of 76.15 (13)° with the pyrrolidine ring (N1/C1-C4) to which it is fused. The mean plane of the cyclohexane ring (C4-C9) and the benzene ring (C11-C16) are almost normal to the pyrrolidine ring with dihedral angles of 88.47 (8) and 77.85 (8)°, respectively.

In the crystal, molecules are linked via a pair of N-H···O hydrogen bonds forming inversion dimers (Table 1). These dimers are linked via a pair of C-H···O hydrogen bonds forming chains along the a axis direction (Table 1).

### Experimental

The synthesis of the title compound is described in Fig. 2. A solution of sulfuryl chloride (0.80 g, 6mmoL) in anhydrous chloroform (10 ml) was added drop wise to a solution of compound 2 (0.90 g, 3mmoL) in anhydrous chloroform (20 ml) at 0 degree and stirred for 10 min. The reaction mixture was allowed to warm to room temperature and stirred at room temperature for 1 h. The reaction mixture was then washed with water (15 ml), saturated sodium bicarbonate (15 ml) and saturated sodium chloride solution and dried over anhydrous Na_2_SO_4_. The solvent was evaporated, and the residual solid was crystallized from ethanol to afford 0.95 g compound 3 as a white solid: yield 94.8%. To a solution of compound 3 (100 mg, 0.30 mmoL) in 2-propanol (10 ml) was added NaBH_4_ (13.6 mg, 0.36 mmoL) at 0 degree. Then the reaction mixture was allowed to room temperature and stirred for 2 h. After removal of the solvent *in vacuo*, 1 N HCl (10 ml) was added to the residue and the whole mixture was extracted with CH_2_Cl_2_ (8 ml τimes 3). The organic layer was washed successively with 3% Na_2_CO_3_ (8 ml) and water (8 ml) and dried over Na_2_SO_4_. Evaporation of the solvent gave a residue, which was purified by flash chromatography on silica gel using a mixture of petroleum ether (boiling point range 60–90 degree) and ethyl acetate (1:1 by volume) as the eluent to afford 32 mg compound 4 as a white solid [yield 35.6%; ESI-MS: 336 (*M*+H)+ (100%)]. Spectroscopic data for the title compound is available in the archived CIF.

### Refinement

The H atoms were included in calculated positions (C–H = 0.93–0.98 Å) and refined as riding, with *U*_iso_(H) = 1.2*U*_eq_(C).

### Crystal data

**Table:**

C_18_H_23_NO_3_	*F*(000) = 648
*M**_r_* = 301.37	*D*_x_ = 1.254 Mg m^−^^3^
Monoclinic, *P*2_1_/*n*	Mo *K*α radiation, λ = 0.71073 Å
Hall symbol: -P 2yn	Cell parameters from 9900 reflections
*a* = 9.1932 (4) Å	θ = 3.0–27.4°
*b* = 9.8139 (4) Å	µ = 0.09 mm^−^^1^
*c* = 17.6979 (7) Å	*T* = 296 K
β = 91.198 (1)°	Block, colourless
*V* = 1596.38 (11) Å^3^	0.53 × 0.38 × 0.36 mm
*Z* = 4	

### Data collection

**Table:**

Rigaku R-AXIS RAPID/ZJUG diffractometer	3629 independent reflections
Radiation source: rotating anode	2407 reflections with *I* > 2σ(*I*)
Graphite monochromator	*R*_int_ = 0.040
Detector resolution: 10.00 pixels mm^-1^	θ_max_ = 27.5°, θ_min_ = 3.0°
ω scans	*h* = −11→11
Absorption correction: multi-scan (*ABSCOR*; Higashi, 1995)	*k* = −12→12
*T*_min_ = 0.946, *T*_max_ = 0.970	*l* = −22→22
15333 measured reflections	

### Refinement

**Table:**

Refinement on *F*^2^	Secondary atom site location: difference Fourier map
Least-squares matrix: full	Hydrogen site location: inferred from neighbouring sites
*R*[*F*^2^ > 2σ(*F*^2^)] = 0.045	H-atom parameters constrained
*wR*(*F*^2^) = 0.122	*w* = 1/[σ^2^(*F*_o_^2^) + (0.0501*P*)^2^ + 0.4587*P*] where *P* = (*F*_o_^2^ + 2*F*_c_^2^)/3
*S* = 1.00	(Δ/σ)_max_ < 0.001
3629 reflections	Δρ_max_ = 0.26 e Å^−^^3^
203 parameters	Δρ_min_ = −0.23 e Å^−^^3^
0 restraints	Extinction correction: *SHELXL97* (Sheldrick, 2008), Fc^*^=kFc[1+0.001xFc^2^λ^3^/sin(2θ)]^-1/4^
Primary atom site location: structure-invariant direct methods	Extinction coefficient: 0.049 (3)

### Special details

**Table:**

Experimental. Spectroscopic data for the title compound: ^1^H NMR (500 MHz, CDCl_3_): 7.28 (s, 1H, Ph—H), 7.21 (s, 1H, Ph—H), 7.11 (s, 1H, Ph—H), 5.57 (s, 1H, –NH–), 3.80 (d, J = 2.65 Hz, 1H, –CH—O—C–), 3.40–3.39 (m, 1H, –CH—O–), 3.37 (s, 3H, –OCH3), 2.35 (s, 3H, Ph—Me), 2.33 (s, 3H, Ph—Me), 2.04–2.00 (m, 1H, Cyclohexane-H1), 1.94–1.82 (m, 4H, Cyclohexane-H4), 1.75–1.62 (m, 3H, Cyclohexane-H3); ^13^C NMR (125 MHz, CDCl_3_): 171.4, 135.4, 133.7, 130.1, 129.8, 128.8, 128.5, 75.3, 64.9, 62.8, 57.0, 55.7, 31.7, 28.2, 27.1, 26.4, 20.8, 19.3.
Geometry. Bond distances, angles etc. have been calculated using the rounded fractional coordinates. All su's are estimated from the variances of the (full) variance-covariance matrix. The cell esds are taken into account in the estimation of distances, angles and torsion angles
Refinement. Refinement of *F*^2^ against ALL reflections. The weighted *R*-factor *wR* and goodness of fit *S* are based on *F*^2^, conventional *R*-factors *R* are based on *F*, with *F* set to zero for negative *F*^2^. The threshold expression of *F*^2^ > σ(*F*^2^) is used only for calculating *R*-factors(gt) *etc*. and is not relevant to the choice of reflections for refinement. *R*-factors based on *F*^2^ are statistically about twice as large as those based on *F*, and *R*- factors based on ALL data will be even larger.

### Fractional atomic coordinates and isotropic or equivalent isotropic displacement parameters (Å^2^)

**Table:**

	*x*	*y*	*z*	*U*_iso_*/*U*_eq_	
O1	0.60742 (14)	0.85489 (13)	0.31242 (7)	0.0625 (4)	
O2	0.42952 (12)	0.79911 (13)	0.47491 (7)	0.0565 (4)	
O3	1.09024 (13)	0.91600 (12)	0.60804 (7)	0.0563 (4)	
N1	0.71200 (14)	0.84251 (13)	0.43139 (7)	0.0448 (4)	
C1	0.60987 (17)	0.81496 (16)	0.37776 (9)	0.0436 (5)	
C2	0.49630 (16)	0.72456 (16)	0.41369 (9)	0.0423 (5)	
C3	0.54729 (17)	0.70679 (17)	0.49249 (9)	0.0468 (5)	
C4	0.69248 (17)	0.77653 (16)	0.50507 (8)	0.0418 (5)	
C5	0.81229 (19)	0.67175 (16)	0.52153 (10)	0.0494 (5)	
C6	0.96044 (18)	0.73875 (18)	0.53488 (10)	0.0520 (6)	
C7	0.95550 (18)	0.84352 (17)	0.59826 (9)	0.0482 (5)	
C8	0.83812 (18)	0.94787 (17)	0.58211 (9)	0.0491 (5)	
C9	0.68976 (18)	0.88081 (18)	0.56940 (9)	0.0499 (5)	
C10	1.2063 (2)	0.8335 (2)	0.63718 (12)	0.0688 (7)	
C11	0.39938 (16)	0.63409 (15)	0.36728 (9)	0.0406 (5)	
C12	0.45709 (17)	0.52733 (16)	0.32516 (9)	0.0444 (5)	
C13	0.3604 (2)	0.44859 (18)	0.28230 (9)	0.0524 (6)	
C14	0.21297 (19)	0.47469 (18)	0.27957 (9)	0.0515 (5)	
C15	0.15504 (17)	0.58089 (18)	0.32083 (9)	0.0479 (5)	
C16	0.25058 (17)	0.65911 (16)	0.36478 (9)	0.0455 (5)	
C17	0.61810 (19)	0.4977 (2)	0.32605 (11)	0.0622 (7)	
C18	−0.00622 (19)	0.6083 (2)	0.31909 (12)	0.0702 (8)	
H1	0.78430	0.89560	0.42300	0.0540*	
H3	0.52710	0.62120	0.51880	0.0560*	
H5A	0.78730	0.61940	0.56590	0.0590*	
H5B	0.81810	0.60930	0.47920	0.0590*	
H6A	1.03190	0.66930	0.54780	0.0620*	
H6B	0.99050	0.78300	0.48870	0.0620*	
H7	0.93360	0.79660	0.64560	0.0580*	
H8A	0.86290	0.99970	0.53750	0.0590*	
H8B	0.83310	1.01070	0.62430	0.0590*	
H9A	0.61800	0.95040	0.55750	0.0600*	
H9B	0.66110	0.83580	0.61550	0.0600*	
H10A	1.17290	0.78150	0.67940	0.1030*	
H10B	1.28570	0.89070	0.65330	0.1030*	
H10C	1.23840	0.77260	0.59840	0.1030*	
H13	0.39640	0.37600	0.25460	0.0630*	
H14	0.15190	0.42050	0.24970	0.0620*	
H16	0.21370	0.73030	0.39330	0.0550*	
H17A	0.63690	0.42140	0.29380	0.0930*	
H17B	0.65020	0.47680	0.37670	0.0930*	
H17C	0.66960	0.57610	0.30820	0.0930*	
H18A	−0.04210	0.60580	0.26780	0.1050*	
H18B	−0.02450	0.69660	0.34030	0.1050*	
H18C	−0.05480	0.54010	0.34810	0.1050*	

### Atomic displacement parameters (Å^2^)

**Table:**

	*U*^11^	*U*^22^	*U*^33^	*U*^12^	*U*^13^	*U*^23^
O1	0.0669 (8)	0.0706 (8)	0.0493 (7)	−0.0175 (6)	−0.0146 (6)	0.0190 (6)
O2	0.0454 (7)	0.0587 (7)	0.0656 (8)	0.0023 (5)	0.0056 (6)	−0.0174 (6)
O3	0.0560 (7)	0.0530 (7)	0.0590 (7)	−0.0094 (6)	−0.0168 (6)	0.0124 (6)
N1	0.0435 (7)	0.0462 (7)	0.0445 (7)	−0.0098 (6)	−0.0046 (6)	0.0091 (6)
C1	0.0428 (8)	0.0416 (8)	0.0460 (8)	−0.0011 (7)	−0.0061 (7)	0.0053 (7)
C2	0.0392 (8)	0.0400 (8)	0.0477 (8)	0.0023 (6)	0.0018 (7)	−0.0008 (7)
C3	0.0500 (9)	0.0447 (8)	0.0457 (9)	−0.0055 (7)	0.0038 (7)	0.0000 (7)
C4	0.0460 (9)	0.0418 (8)	0.0376 (8)	−0.0044 (7)	−0.0015 (6)	0.0031 (6)
C5	0.0621 (11)	0.0392 (8)	0.0467 (9)	0.0000 (7)	−0.0062 (8)	0.0012 (7)
C6	0.0525 (10)	0.0466 (9)	0.0565 (10)	0.0056 (8)	−0.0084 (8)	−0.0006 (8)
C7	0.0546 (10)	0.0473 (9)	0.0422 (8)	−0.0091 (8)	−0.0077 (7)	0.0068 (7)
C8	0.0597 (10)	0.0428 (8)	0.0447 (8)	−0.0027 (8)	−0.0033 (7)	−0.0049 (7)
C9	0.0516 (10)	0.0507 (9)	0.0473 (9)	−0.0008 (8)	0.0013 (7)	−0.0048 (7)
C10	0.0639 (12)	0.0686 (12)	0.0728 (13)	−0.0008 (10)	−0.0226 (10)	0.0132 (10)
C11	0.0376 (8)	0.0394 (8)	0.0447 (8)	−0.0024 (6)	0.0008 (6)	0.0038 (7)
C12	0.0436 (9)	0.0453 (9)	0.0443 (8)	0.0033 (7)	0.0020 (7)	0.0015 (7)
C13	0.0612 (11)	0.0516 (9)	0.0444 (9)	0.0018 (8)	0.0022 (8)	−0.0059 (8)
C14	0.0548 (10)	0.0572 (10)	0.0421 (8)	−0.0102 (8)	−0.0050 (7)	0.0001 (8)
C15	0.0407 (8)	0.0556 (10)	0.0474 (9)	−0.0055 (7)	−0.0013 (7)	0.0081 (8)
C16	0.0419 (9)	0.0428 (8)	0.0519 (9)	0.0001 (7)	0.0028 (7)	0.0002 (7)
C17	0.0528 (11)	0.0624 (11)	0.0715 (12)	0.0123 (9)	0.0046 (9)	−0.0057 (10)
C18	0.0423 (10)	0.0888 (15)	0.0793 (14)	−0.0067 (10)	−0.0036 (9)	−0.0021 (12)

### Geometric parameters (Å, º)

**Table:**

O1—C1	1.221 (2)	C15—C16	1.392 (2)
O2—C2	1.454 (2)	C15—C18	1.506 (2)
O2—C3	1.441 (2)	C3—H3	0.9800
O3—C7	1.436 (2)	C5—H5A	0.9700
O3—C10	1.427 (2)	C5—H5B	0.9700
N1—C1	1.348 (2)	C6—H6A	0.9700
N1—C4	1.4703 (19)	C6—H6B	0.9700
N1—H1	0.8600	C7—H7	0.9800
C1—C2	1.520 (2)	C8—H8A	0.9700
C2—C3	1.472 (2)	C8—H8B	0.9700
C2—C11	1.492 (2)	C9—H9A	0.9700
C3—C4	1.512 (2)	C9—H9B	0.9700
C4—C9	1.532 (2)	C10—H10A	0.9600
C4—C5	1.530 (2)	C10—H10B	0.9600
C5—C6	1.526 (2)	C10—H10C	0.9600
C6—C7	1.523 (2)	C13—H13	0.9300
C7—C8	1.511 (2)	C14—H14	0.9300
C8—C9	1.527 (2)	C16—H16	0.9300
C11—C12	1.397 (2)	C17—H17A	0.9600
C11—C16	1.390 (2)	C17—H17B	0.9600
C12—C17	1.508 (2)	C17—H17C	0.9600
C12—C13	1.391 (2)	C18—H18A	0.9600
C13—C14	1.379 (3)	C18—H18B	0.9600
C14—C15	1.385 (2)	C18—H18C	0.9600
			
C2—O2—C3	61.14 (10)	C4—C5—H5B	109.00
C7—O3—C10	113.52 (13)	C6—C5—H5A	109.00
C1—N1—C4	116.10 (13)	C6—C5—H5B	109.00
C4—N1—H1	122.00	H5A—C5—H5B	108.00
C1—N1—H1	122.00	C5—C6—H6A	109.00
O1—C1—C2	125.77 (15)	C5—C6—H6B	109.00
N1—C1—C2	107.27 (13)	C7—C6—H6A	109.00
O1—C1—N1	126.96 (15)	C7—C6—H6B	109.00
O2—C2—C11	116.94 (12)	H6A—C6—H6B	108.00
O2—C2—C1	108.82 (12)	O3—C7—H7	109.00
O2—C2—C3	58.99 (10)	C6—C7—H7	109.00
C3—C2—C11	128.73 (14)	C8—C7—H7	109.00
C1—C2—C11	121.65 (14)	C7—C8—H8A	109.00
C1—C2—C3	104.93 (13)	C7—C8—H8B	109.00
O2—C3—C4	113.84 (13)	C9—C8—H8A	109.00
C2—C3—C4	110.39 (13)	C9—C8—H8B	109.00
O2—C3—C2	59.87 (10)	H8A—C8—H8B	108.00
N1—C4—C9	111.67 (13)	C4—C9—H9A	109.00
N1—C4—C3	101.10 (12)	C4—C9—H9B	109.00
N1—C4—C5	111.35 (13)	C8—C9—H9A	109.00
C5—C4—C9	109.29 (13)	C8—C9—H9B	109.00
C3—C4—C5	110.70 (13)	H9A—C9—H9B	108.00
C3—C4—C9	112.57 (13)	O3—C10—H10A	109.00
C4—C5—C6	112.11 (13)	O3—C10—H10B	110.00
C5—C6—C7	111.38 (14)	O3—C10—H10C	109.00
O3—C7—C8	107.34 (13)	H10A—C10—H10B	109.00
O3—C7—C6	112.60 (13)	H10A—C10—H10C	109.00
C6—C7—C8	110.52 (13)	H10B—C10—H10C	109.00
C7—C8—C9	111.60 (14)	C12—C13—H13	119.00
C4—C9—C8	111.66 (13)	C14—C13—H13	119.00
C2—C11—C16	119.24 (14)	C13—C14—H14	120.00
C2—C11—C12	120.73 (13)	C15—C14—H14	120.00
C12—C11—C16	120.01 (14)	C11—C16—H16	119.00
C11—C12—C17	121.51 (14)	C15—C16—H16	119.00
C11—C12—C13	117.53 (15)	C12—C17—H17A	109.00
C13—C12—C17	120.96 (15)	C12—C17—H17B	109.00
C12—C13—C14	122.16 (16)	C12—C17—H17C	109.00
C13—C14—C15	120.64 (16)	H17A—C17—H17B	109.00
C14—C15—C16	117.67 (15)	H17A—C17—H17C	109.00
C14—C15—C18	120.83 (15)	H17B—C17—H17C	110.00
C16—C15—C18	121.48 (15)	C15—C18—H18A	109.00
C11—C16—C15	121.98 (15)	C15—C18—H18B	109.00
O2—C3—H3	120.00	C15—C18—H18C	109.00
C2—C3—H3	119.00	H18A—C18—H18B	109.00
C4—C3—H3	120.00	H18A—C18—H18C	109.00
C4—C5—H5A	109.00	H18B—C18—H18C	109.00
			
C2—O2—C3—C4	100.60 (14)	O2—C3—C4—N1	−60.56 (15)
C3—O2—C2—C1	−96.47 (14)	O2—C3—C4—C5	−178.64 (13)
C3—O2—C2—C11	120.88 (16)	C2—C3—C4—C5	−113.55 (15)
C10—O3—C7—C8	169.18 (14)	C2—C3—C4—N1	4.53 (16)
C10—O3—C7—C6	−68.97 (18)	C3—C4—C5—C6	−179.75 (13)
C4—N1—C1—C2	2.84 (18)	N1—C4—C5—C6	68.63 (17)
C4—N1—C1—O1	−177.41 (15)	C3—C4—C9—C8	178.88 (13)
C1—N1—C4—C5	113.01 (15)	C5—C4—C9—C8	55.45 (17)
C1—N1—C4—C9	−124.52 (15)	C9—C4—C5—C6	−55.21 (18)
C1—N1—C4—C3	−4.60 (17)	N1—C4—C9—C8	−68.20 (17)
N1—C1—C2—O2	62.13 (16)	C4—C5—C6—C7	55.95 (18)
O1—C1—C2—C11	22.9 (2)	C5—C6—C7—C8	−55.29 (18)
O1—C1—C2—O2	−117.63 (17)	C5—C6—C7—O3	−175.32 (13)
O1—C1—C2—C3	−179.43 (16)	O3—C7—C8—C9	179.04 (12)
N1—C1—C2—C11	−157.31 (14)	C6—C7—C8—C9	55.90 (17)
N1—C1—C2—C3	0.32 (17)	C7—C8—C9—C4	−56.98 (17)
O2—C2—C11—C16	22.8 (2)	C2—C11—C12—C13	−178.99 (15)
C1—C2—C11—C12	63.6 (2)	C2—C11—C12—C17	1.2 (2)
C1—C2—C3—C4	−3.17 (17)	C16—C11—C12—C13	−0.6 (2)
C3—C2—C11—C12	−88.3 (2)	C16—C11—C12—C17	179.58 (15)
C3—C2—C11—C16	93.3 (2)	C2—C11—C16—C15	178.10 (15)
C1—C2—C3—O2	103.25 (13)	C12—C11—C16—C15	−0.3 (2)
C11—C2—C3—C4	152.30 (15)	C11—C12—C13—C14	1.2 (2)
O2—C2—C11—C12	−158.82 (14)	C17—C12—C13—C14	−179.01 (16)
C1—C2—C11—C16	−114.81 (17)	C12—C13—C14—C15	−0.8 (3)
O2—C2—C3—C4	−106.43 (14)	C13—C14—C15—C16	−0.1 (2)
C11—C2—C3—O2	−101.28 (17)	C13—C14—C15—C18	−178.94 (16)
O2—C3—C4—C9	58.71 (17)	C14—C15—C16—C11	0.7 (2)
C2—C3—C4—C9	123.80 (14)	C18—C15—C16—C11	179.49 (16)

### Hydrogen-bond geometry (Å, º)

**Table:**

*D*—H···*A*	*D*—H	H···*A*	*D*···*A*	*D*—H···*A*
N1—H1···O3^i^	0.86	2.25	3.0760 (17)	160
C9—H9*A*···O2^ii^	0.97	2.56	3.413 (2)	147

Symmetry codes: (i) −*x*+2, −*y*+2, −*z*+1; (ii) −*x*+1, −*y*+2, −*z*+1.
